# The risk of depressive symptoms in offspring exposed to prenatal alcohol and tobacco use: evidence from a population-based longitudinal study

**DOI:** 10.1192/j.eurpsy.2022.667

**Published:** 2022-09-01

**Authors:** B. Duko, G. Pereira, K. Betts, R. Tait, J. Newnham, R. Alati

**Affiliations:** 1 Curtin University, Curtin School Of Population Health, Perth, Australia; 2 Curtin University, National Drug Research Institute (ndri), Perth, Australia; 3 The University of Western Australia, Division Of Obstetrics And Gynecology, Faculty Of Health And Medical Sciences, Perth, Australia

**Keywords:** tobacco, offspring, alcohol, depressive symptoms

## Abstract

**Introduction:**

Evidence from epidemiological studies indicated that intrauterine exposure to alcohol and tobacco is linked with a number of adverse outcomes in offspring. However, few studies have linked prenatal alcohol and tobacco exposures to offspring depressive symptoms with mixed results.

**Objectives:**

The objective of this study was to examine the link between maternal prenatal alcohol and tobacco exposures and depressive symptoms in offspring.

**Methods:**

Using data from the Raine Study, a prospective multigenerational observational study, we examined the associations between maternal prenatal alcohol and tobacco use and the risk of depressive symptoms in offspring at age 17 years (N=1168). Depressive symptoms in offspring were measured using the Beck Depression Inventory for Youth. Log-binomial regression was used to estimate relative risk (RR) for associations between exposures and outcome. To better investigate the role of potential confounders, risk factors were sequentially added as adjustment variables in separate models.

**Results:**

After adjustment for potential confounders, depressive symptoms in offspring remained related to maternal alcohol use of six or more standard drinks per week during the first trimester of pregnancy [RR 1.59 (95% CI: 1.11-2.26)]. Further, the risk of depressive symptoms was 50% higher for offspring exposed to prenatal tobacco use when compared to non-exposed. The Associations did not appear to be mediated by the effects of prenatal alcohol and tobacco use on adverse pregnancy outcomes.

**Conclusions:**

Early screening and prevention of these exposures could possibly reduce depressive symptoms in offspring. Moreover, future examinations such as Mendelian Randomization that allow a stronger causal inference is warranted.

**Disclosure:**

No significant relationships.

